# Dose and schedule-finding study of oral topotecan and weekly cisplatin in patients with recurrent ovarian cancer

**DOI:** 10.1054/bjoc.2001.2014

**Published:** 2001-10

**Authors:** H Gelderblom, A Sparreboom, M J A de Jonge, W J Loos, E Wilms, M A Mantel, B Hennis, I Camlett, J Verweij, M E L van der Burg

**Affiliations:** Department of Medical Oncology, Department of Pharmacy, Rotterdam Cancer Institute (Daniel den Hoed Kliniek) and University Hospital Rotterdam, Rotterdam, AE, 3075, The Netherlands; SmithKline Beecham Pharmaceuticals, Zeist, The Netherlands; Glaxo SmithKline Beecham Pharmaceuticals, New Frontiers Science Park (South), Third Avenue, Harlow, Essex, CM19 5AW, UK; Department of Clinical Oncology, Leiden University Medical Centre, KI-p, PO Box 9600, Leiden, RC, 2300, The Netherlands

**Keywords:** cisplatin, oral topotecan, cremophor EL, ovarian cancer

## Abstract

Both weekly cisplatin chemotherapy and single agent topotecan have proven to be effective in recurrent ovarian cancer. Preclinical data show synergism between cisplatin and topotecan. Side effects for this combination are drug sequence dependent and predominantly haematologic. Since preclinical data suggest that Cremophor EL (CrEL), the formulation vehicle of paclitaxel, has a protective effect on haematological toxicity of cisplatin, CrEL was added to the combination cisplatin and topotecan. In this phase I study, escalating doses of oral topotecan administered on day 1, 2, 8, 9, 15, 16, 29, 30, 36, 37, 43, 44 were combined with weekly cisplatin 70 mg m^−2^d^−1^on day 1, 8, 15, 29, 36, 43 (scheme A) or with the presumably less myelotoxic sequence weekly cisplatin day 2, 9, 16, 30, 37, 44 (scheme B). In scheme C, CrEL 12 ml was administered prior to cisplatin in the sequence of Scheme A. 18 patients have received a total of 85 courses. In scheme A 4/10 patients, all treated with topotecan 0.45 mg m^−2^d^−1^, experienced DLT: 1 patient had vomiting grade 4, 1 patient had grade 4 neutropenia >5 days, 1 patient had >2 weeks delay due to thrombocytopenia and 1 patient due to neutropenia. Both patients in scheme B (topotecan 0.45 mg m^−2^d^−1^) had DLT due to a delay > 2 weeks because of prolonged haematological toxicity. No DLT was observed in the first 3 patients in scheme C (topotecan 0.45 mg m^−2^d^−1^). However, 2 out of 3 patients treated at dose level topotecan 0.60 mg m^−2^d^−1^in scheme C experienced DLT due to >2 weeks delay because of persistent thrombocytopenia or neutropenia. We conclude that there is a modest clinical effect of CrEL on haematological toxicity for this cisplatin-based combination regimen, which seems to reduce these side effects but does not really enable an increase of the oral topotecan dose. © 2001 Cancer Research Campaign  http://www.bjcancer.com


British Journal of Cancer (2001) 85(8), 1124–1129
© 2001 Cancer Research Campaign
doi: 10.1054/ bjoc.2001.2014, available online at http://www.idealibrary.com on IDEAL
http://www.bjcancer.com

### Dose and schedule-finding study of oral topotecan and weekly cisplatin in patients with recurrent ovarian cancer

**H Gelderblom[1,5], A Sparreboom[1], MJA de Jonge[1], WJ Loos[1], E Wilms[2], MA Mantel[1], B Hennis[3], I Camlett[4], J Verweij[1] and MEL van der Burg[1]**

[1]Department of Medical Oncology and [2]Department of Pharmacy, Rotterdam Cancer Institute (Daniel den Hoed Kliniek) and University Hospital Rotterdam, 3075 AE Rotterdam, The Netherlands; [3]SmithKline Beecham Pharmaceuticals, Zeist, The Netherlands; [4]Glaxo SmithKline Beecham Pharmaceuticals, New Frontiers Science Park (South), Third Avenue, Harlow, Essex CM19 5AW, UK; [5]Leiden University Medical Centre, Department of Clinical Oncology, KI-p, PO Box 9600, 2300 RC Leiden, The Netherlands

**Summary** Both weekly cisplatin chemotherapy and single agent topotecan have proven to be effective in recurrent ovarian cancer. Preclinical data show synergism between cisplatin and topotecan. Side effects for this combination are drug sequence dependent and predominantly haematologic. Since preclinical data suggest that Cremophor EL (CrEL), the formulation vehicle of paclitaxel, has a protective effect on haematological toxicity of cisplatin, CrEL was added to the combination cisplatin and topotecan. In this phase I study, escalating doses of oral topotecan administered on day 1, 2, 8, 9, 15, 16, 29, 30, 36, 37, 43, 44 were combined with weekly cisplatin 70 mg $m^{-2}$ $d^{-1}$ on day 1, 8, 15, 29, 36, 43 (scheme A) or with the presumably less myelotoxic sequence weekly cisplatin day 2, 9, 16, 30, 37, 44 (scheme B). In scheme C, CrEL 12 ml was administered prior to cisplatin in the sequence of Scheme A. 18 patients have received a total of 85 courses. In scheme A 4/10 patients, all treated with topotecan 0.45 mg $m^{-2}$ $d^{-1}$, experienced DLT: 1 patient had vomiting grade 4, 1 patient had grade 4 neutropenia >5 days, 1 patient had >2 weeks delay due to thrombocytopenia and 1 patient due to neutropenia. Both patients in scheme B (topotecan 0.45 mg $m^{-2}$ $d^{-1}$) had DLT due to a delay > 2 weeks because of prolonged haematological toxicity. No DLT was observed in the first 3 patients in scheme C (topotecan 0.45 mg $m^{-2}$ $d^{-1}$). However, 2 out of 3 patients treated at dose level topotecan 0.60 mg $m^{-2}$ $d^{-1}$ in scheme C experienced DLT due to >2 weeks delay because of persistent thrombocytopenia or neutropenia. We conclude that there is a modest clinical effect of CrEL on haematological toxicity for this cisplatin-based combination regimen, which seems to reduce these side effects but does not really enable an increase of the oral topotecan dose. © 2001 Cancer Research Campaign http://www.bjcancer.com

**Keywords**: cisplatin; oral topotecan; cremophor EL; ovarian cancer

Ovarian cancer is the most common cause of death among patients with gynaecological malignancies (Parker et al, 1997). At the time of presentation the majority of patients have advanced disease, which is not amenable to cure by surgery alone. The standard treatment is a multimodality approach, consisting of cytoreductive surgery and platinum/paclitaxel combination chemotherapy (McGuire and Ozols, 1998). Salvage chemotherapy may result in prolonged secondary remissions with alleviation of symptoms and improvement of the quality of life. The response to salvage chemotherapy is related to the therapy-free interval. The longer this interval, the greater the probability of response to retreatment with platinum-based regimens. Dose-intense combination therapy might increase the response rates and the disease and overall survival. There is no standard therapy for patients with early recurrent ovarian cancer. Studies with new agents, including topotecan, have shown activity (Creemers et al, 1994; Swisher et al, 1997; ten Bokkel Huinink et al, 1997). Cisplatin administered weekly at a dose of 70 mg $m^{-2}$ in combination with either continuous oral etoposide or with weekly i.v. paclitaxel to patients with progressive or recurrent ovarian cancer yielded a high dose intensity with response rates up to 84% in second-line treatment (van der Burg et al, 1996, 1998).

*Received 16 February 2001*
*Revised 11 June 2001*
*Accepted 5 July 2001*

*Correspondence to:* H Gelderblom

Since there is preclinical evidence of synergistic action between cisplatin and topotecan (Chou et al, 1994; Romanelli et al, 1998) and both agents are active in ovarian cancer, while their toxicity profile is mostly non-overlapping, we investigated a dose-intense weekly combination of these agents in recurrent ovarian cancer. We administered topotecan orally since preclinical studies suggested that prolonged exposure, as can be achieved more conveniently by oral administration, might result in higher antitumour activity (Hochster et al, 1994).

Combining topotecan with i.v. cisplatin in more conventional 3-weekly schedules required considerable dose reduction of topotecan as compared to the single agent dose, even with G-CSF support (Miller et al, 1994). Dose-limiting toxicities (DLT) consisted mainly of haematological toxicity, which was sequence dependent, with topotecan following cisplatin as the most haematotoxic sequence (Rowinsky et al, 1996; de Jonge et al, 2000). Whether drug sequencing clinically is also relevant for antitumour activity is as yet unknown. To evaluate the sequence-dependent effects we administered the oral topotecan 2 days a week preceding weekly cisplatin or following cisplatin.

The third schedule was based on the preclinical observation that CrEL protected from cisplatin-induced haematological toxicity (Ma et al, 1996; de Vos et al, 1997; Badary et al, 2000). In these studies CrEL inhibited DNA adduct formation and the intracellular accumulation of cisplatin in human leukocytes, and in mice CrEL protected from cisplatin induced myelotoxicity. We might have observed this effect clinically in a study with weekly cisplatin preceded by paclitaxel (van der Burg et al, 1998). In this study an unexpected high dose of weekly paclitaxel 90 mg $m^{-2}$ could be safely combined with weekly cisplatin 70 mg $m^{-2}$. Therefore, to evaluate the possibility of an increase of the oral topotecan dose i.v. CrEL was administered prior to the weekly cisplatin infusions in the third cohort of the current study.

### PATIENTS AND METHODS

### Patients selection

Patients with progressive or recurrent ovarian cancer were eligible for this phase I study. In case of prior treatment with a weekly cisplatin-based regimen, a minimal progression-free interval of 3 months after the completion of the weekly cisplatin schedule, was mandatory. Other eligibility criteria included: WHO performance status 0–2, evaluable disease, no more than 2 prior chemotherapy regimens, no chemotherapy, hormonal or radiotherapy for at least 4 weeks prior to entry in the study, no signs of bowel obstruction, neutrophils ≥ 1.5 × $10^9$ $l^{-1}$, platelet count ≥ 100 × $10^9$ $l^{-1}$, total bilirubin < 1.25 times the upper limit of normal, creatinine clearance > 60 ml $min^{-1}$, peripheral neurotoxicity ≤ grade 1, no condition precluding adequate intake of oral topotecan, and no prior therapy with a topoisomerase I inhibitor. The study was approved by the institution's medical ethics committee and all patients signed written informed consent prior to entry in the study.

### Treatment assessment

Before therapy a complete medical history was taken and a physical examination was performed. A complete blood count (CBC) including white blood cell (WBC) differential, and serum biochemistry, which involved sodium, potassium, calcium, phosphorus, urea, creatinine, total protein, albumin, total bilirubin, alkaline phosphatase, aspartate aminotransferase (ASAT), alanine transferase (ALAT), γ-glutamyl transferase, glucose and uric acid, were performed, as was 24-hour creatinine clearance. During the induction regimen, weekly evaluations included history, physical examination, toxicity assessment according to the National Cancer Institute Common Toxicity Criteria (NCI-CTC) version 2.0, and serum chemistry. CBC was determined twice weekly. Tumour evaluation according to the World Health Organisation (WHO) criteria for response was performed before and after the induction regimen and every 2 courses during the maintenance regimen.

### Drug administration

Cisplatin (Platosin) was supplied as a powder by Pharmachemie (Haarlem, the Netherlands). All patients received cisplatin dissolved in 250 ml of hypertonic saline (3% (w/v) sodium chloride) as a 3-h infusion on a weekly base during the induction regimen and 3-weekly during the maintenance regimen. Topotecan capsules containing either 0.25 or 1.00 mg of the active compound were supplied by Smith Kline Beecham Pharmaceuticals (Harlow, UK). Topotecan was administered orally immediately at the start of the cisplatin infusion and the following day (scheme A and C) or the previous day (scheme B) during the induction regimen and for 5 consecutive days during the maintenance regimen (days 1–5) on an empty stomach, at least 10 min before meals. CrEL was obtained from Duchefa (Haarlem, the Netherlands), and administered through a polyvinylchloride-free infusion system in 3 h before the cisplatin infusions in scheme C.

In all patients standard pre-medication consisted of ondansetron (8 mg i.v.) combined with dexamethasone (10 mg i.v.), and an additional 2 mg of clemastine in scheme C. To avoid cisplatin-induced renal damage, the administration of cisplatin was preceded by the infusion of 1 l of a mixture of 2.5% (w/v) dextrose and 0.45% (w/v) sodium chloride over 4 h, and followed by another 3 l with the addition of 20 mM potassium chloride and 2 g $l^{-1}$ magnesium sulfate applied over 16 h. Further anti-emetic therapy consisted of oral dexamethason 3 mg twice daily and ondansetron 8 mg twice daily on the first 2 days following cisplatin infusion, followed by oral metoclopramide or domperidon as needed.

### Treatment and dose escalation

Patients were treated with an induction regimen consisting of oral topotecan day 1, 2, 8, 9, 15, 16, 29, 30, 36, 37, 43, 44 in combination with weekly cisplatin 70 mg $m^{-2}$ $d^{-1}$ on day 1, 8, 15, 29, 36, 43 (scheme A) or the same administration scheme of topotecan in the presumably less myelotoxic sequence with weekly cisplatin on day 2, 9, 16, 30, 37, 44 (scheme B). In scheme C CrEL (12 ml) was administered prior to cisplatin using the sequence of scheme A. The dose of CrEL was similar to that administered with paclitaxel at a dose of 90 mg $m^{-2}$, as used in our weekly cisplatin/paclitaxel schedule.[8] All schemes are outlined in Figure 1. The topotecan starting dose was 0.45 mg $m^{-2}$ $d^{-1}$, a dose deduced from previous phase I studies with single agent topotecan, and our recently completed study with oral topotecan given daily 5 times every 3 weeks in combination with 3-weekly cisplatin (de Jonge et al, 2000). Topotecan dose was escalated depending on the observed toxicity. Weekly treatment was postponed if the neutrophil count had not recovered to ≥ 1.0 × $10^9$ $l^{-1}$ and the platelet count to ≥ 100 × $10^9$ $l^{-1}$ on the day of retreatment. If the treatment was delayed, haematology was assessed twice a week to enable treatment re-start as soon as possible. Dose-limiting toxicity (DLT) was defined as

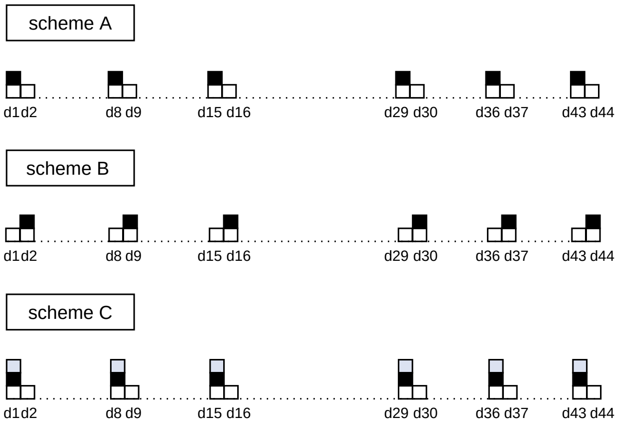

**Figure 1** Outline of the different induction regimens: scheme A, B and C. Cisplatin administration (closed symbols), oral topotecan administration (open symbols) and CrEL administration (grey symbols)

*© 2001 Cancer Research Campaign*

NCI-CTC version 2.0 grade 4 neutropenia lasting for 5 days or more, or complicated with fever requiring hospitalisation, grade 4 thrombocytopenia and/or non-haematological toxicity ≥ grade 3, excluding nausea. Treatment delay of more than 2 weeks due to toxicity was also considered DLT. Dose (de-)escalation, if any, was based on the toxicities observed at previous dose levels. In all patients response evaluation took place after completion of the induction regimen. In case of response or stable disease it was left to the judgement of the treating physician to proceed with a maintenance therapy consisting of cisplatin and/or oral topotecan in a 3-weekly schedule.

### Dose modifications

If neutrophils were $< 1.0 \times 10^9$ $l^{-1}$ and/or platelet count $\leq 100 \times 10^9$ $l^{-1}$ on day 8, 15, 29, 36, 43 or preceding each maintenance course, treatment was delayed until recovery.

Prior to the first course creatinine clearance should exceed 60 ml $min^{-1}$, as stated in the inclusion criteria. If the creatinine clearance was impaired due to dehydration or insufficient intake of fluids, prehydration was intensified followed by new creatinine clearance measurements. If the creatinine clearance after additional prehydration remained < 50 ml $min^{-1}$ during the induction regimen, cisplatin was withdrawn from the combination regimen.

### Sample collection and drug analysis

Blood samples for pharmacokinetics of cisplatin and topotecan were obtained on the day of cisplatin infusion during the first 3 weekly administrations of the induction regimen in scheme C. All blood samples were obtained and analysed as described previously (de Jonge et al, 2000).

### RESULTS

18 patients entered this study between March 1999 and May 2000. Patient characteristics are listed in Table 1. All patients had progressive or recurrent ovarian cancer after prior cisplatin-based chemotherapy and were eligible. The median WHO performance status was 1 (range 0–2), and age 51 years (range 34–68). The median cisplatin-free interval of 7 months (range 0–17) was short, rendering a poor prognosis. A total of 85 weekly induction administrations (cycles) were given. Overall, only 7 patients received all 6 weekly induction administrations: 2 patients were withdrawn because of an allergic reaction to cisplatin infusion during the 4th administration, 7 patients could not complete all 6 induction administrations due to haematological toxicity, 1 patient was not able to receive her 6th induction administration due to electrolyte disturbances related to the weekly platinum infusions, and 1 patient went off study after the 4th cycle due to early progressive disease.

**Table 1** Patient characteristics

| Characteristic | | No. of patients |
|---|---|---|
| No. of patients entered | | 18 |
| No. of patients assessable | | 18 |
| Cisplatin free interval, months | | |
| < 6 | 7 | |
| 6–12 | 8 | |
| > 12 | 3 | |
| Age (years) | | |
| Median | 51 | |
| Range | 34–68 | |
| WHO performance status | | |
| 0 | | 9 |
| 1 | | 7 |
| 2 | | 2 |

In scheme A, 10 patients received cisplatin 70 mg $m^{-2}$ $d^{-1}$ on day 1 followed by oral topotecan at 0.45 mg $m^{-2}$ $d^{-1}$ on day 1 and 2. In scheme B, a total of 2 patients were treated at the same dose level in the reverse sequence. In scheme C, 3 patients were treated at the initial dose level of topotecan at 0.45 mg $m^{-2}$ $d^{-1}$ on day 1 and 2 and another 3 patients at 0.6 mg $m^{-2}$ $d^{-1}$, all preceded by CrEL and cisplatin on day 1.

Haematological toxicity and in one case vomiting were the dose-limiting toxicities (DLTs) of the induction regimen. In scheme A 4 out of 10 patients experienced DLT, in scheme B 2 out of 2 and in scheme C no DLTs were observed at the initial dose level and in 2 out of 3 at the higher dose level. The toxicity of the induction regimen A, B and C will be described separately in more detail in the following paragraphs. The main toxicity of the maintenance regimen was uncomplicated myelotoxicity.

### Haematological toxicity

The haematological toxicity observed during the induction regimen is shown in Table 2. In scheme A, 3 patients were judged as having DLT for haematological reasons: 1 patient had her 3rd weekly cisplatin administration (cycle) postponed more than 2 weeks due to persistent thrombocytopenia, 1 patient developed grade 4 neutropenia lasting for 5 days or more after cycle 3 and 1 patient had grade 4 thrombocytopenia, following cycle 3. In scheme B both patients had DLT based on delay of their 4th cycle by more than 2 weeks because of prolonged neutropenia (1 patient) or neutro-and thrombocytopenia (1 patient). Both patients continued induction treatment with weekly cisplatin monotherapy. At the first dose level in scheme C, with the addition of CrEL, no

**Table 2** Haematological toxicity (worst per cycle)

| Scheme | Topotecan mg $m^{-2}$ $day^{-1}$ | No. of patients/cycles | Leukocytopenia | | Neutropenia | | Thrombocytopenia | |
|---|---|---|---|---|---|---|---|---|
| | | | 3 | 4 | 3 | 4 | 3 | 4 |
| A | 0.45 | 10/43 | 2 | 1 | 5 | 4 | 3 | 1 |
| B | 0.45 | 2/6 | 1 | 0 | 1 | 1 | 0 | 1 |
| C | 0.45 | 3/18 | 0 | 0 | 1 | 1 | 0 | 0 |
| C[a] | 0.60 | 3/18 | 1 | 0 | 1 | 1 | 2 | 0 |

[a]Patients who had their topotecan dose reduced to 0.45 mg $m^{-2}$ $d^{-1}$ due to earlier toxicity were included in this table.

 *© 2001 Cancer Research Campaign*

**Table 3** Non-haematological toxicity (worst per cycle)

| Scheme | Topotecan mg $m^{-2}$ $d^{-1}$ | No. of patients/cycles | Nausea grade | | | Vomiting grade | | | |
|---|---|---|---|---|---|---|---|---|---|
| | | | 1 | 2 | 3 | 1 | 2 | 3 | 4 |
| A | 0.45 | 10/43 | 15 | 11 | 0 | 7 | 11 | 0 | 1 |
| B | 0.45 | 2/6 | 2 | 4 | 0 | 1 | 4 | 0 | 0 |
| C | 0.45 | 3/18 | 4 | 2 | 0 | 2 | 2 | 0 | 0 |
| C[a] | 0.60 | 3/18 | 7 | 2 | 1 | 6 | 1 | 0 | 0 |

[a]Patients who had their topotecan dose reduced to 0.45 mg $m^{-2}$ $d^{-1}$ due to earlier toxicity were included in this table.

DLTs were observed, rendering this dose level feasible. Only 1 grade 4 neutropenia was observed and all patients completed the weekly cisplatin on time. However, in scheme C, at the dose of topotecan at 0.6 mg $m^{-2}$ $d^{-1}$, again DLT was encountered in 2 out of 3 patients. The 3rd cycle was delayed by more than 2 weeks because of thrombocytopenia in 1 patient and thrombocytopenia followed by neutropenia in another patient. The MTD in scheme C was determined as topotecan at 0.45 mg $m^{-2}$ $d^{-1}$ (days 1 and 2) combined with CrEL 12 ml and cisplatin at 70 mg $m^{-2}$ $week^{-1}$.

In scheme A, B, C first dose level and C highest dose level, the percentage of cycles associated with grade 3 or 4 neutropenia were respectively: 21, 33, 11 and 11%, but none of the neutropenic periods was complicated by fever. Thrombocytopenia grade 3 or 4 was observed in only a limited number of cycles (9, 17, 0 and 6%), all without bleeding, only 2 patients received a prophylactic platelet transfusion. Mild (grade 1 or 2) anaemia occurred in almost all patients, only 3 patients (1 in scheme A and 2 in scheme C) had grade 3 anaemia. A total of 20 blood transfusions were administered during induction chemotherapy (8 in scheme A, 4 in scheme B and 8 in scheme C). 3 patients received erythropoietine injections during the last induction cycles.

### Non-haematological toxicity

Nausea and vomiting were the most frequently reported non-haematological side effects (data shown in Table 3). One patient in scheme A was judged as having DLT because of grade 4 vomiting 1 week after cycle 6. The event was only possibly related to the chemotherapy, because she had experienced these symptoms once prior to treatment.

Other side effects included fatigue (grade 1 in 28%, grade 2 in 11%), alopecia in 22%, tinnitus in 17% and nephrotoxicity grade 1 in 28%. Mild headache was reported in 50% of patients, but this might also partially be related to the administration of ondansetron.

2 patients were withdrawn from the study because of an allergic reaction to cisplatin. These patients continued treatment with oral topotecan monotherapy.

### Antitumour activity

3 patients were not assessable for response due to early discontinuation of the induction regimen for reasons other than progression of the disease. 7 patients had early response evaluation after having received more than 3 weekly cycles. Of the 15 patients evaluable for response after the weekly induction regimen, 9 patients (60%) reached a partial response, another 5 (33%) had stable disease, only 1 patient (7%) had early progressive disease. Due to the various maintenance treatments and the small sample size, assessment of the overall response and response duration is not really possible.

### Pharmacokinetics

Full kinetic data following the administration of cisplatin and topotecan during the first 3 cycles were obtained from 6 patients in scheme C. Cisplatin and topotecan pharmacokinetic data (apparent clearance of unbound platinum fraction of 32.2 ± 24.8 l $h^{-1}$ $m^{-2}$ (mean ± SD), cisplatin unbound/bound ratio of 0.082 ± 0.045, apparent oral topotecan clearance of 142 ± 95.7 l $h^{-1}$ $m^{-2}$ and topotecan lactone to total drug AUC ratio of 0.36 ± 0.05) were comparable to single agent data (Gerrits et al, 1998). The variability in $C_{max}$ and AUC for the 6 patients who had full kinetic data were 4.97–41.8% and 28.7–32.2%, respectively, which is also in agreement with earlier data (Gerrits et al, 1998; Gelderblom et al, 2000a). These findings indicate that there was no cisplatin/-topotecan/CrEL pharmacokinetic drug interaction.

### DISCUSSION

This study, using weekly cisplatin, with and without CrEL, in combination with oral topotecan in different sequences, was based on a number of (pre-)clinical observations: (a) combining cisplatin and topotecan, 2 potentially active agents in ovarian cancer in a dose-dense scheme, is attractive given their preclinical synergism; (b) Dose-dense weekly cisplatin combination chemotherapy regimens seem to yield improved response rates in recurrent ovarian cancer; (c) Administration of oral topotecan enables prolonged systemic exposure, which is more effective in pre-clinical models and (d) single agent oral topotecan is as effective as i.v., with less grade 4 neutropenia in advanced ovarian cancer (Gore et al, 1998), (e) while patients have a preference for oral chemotherapy when equally effective (Liu et al, 1997). In order to study drug-sequence-dependent haematological effects for the combination cisplatin and i.v. topotecan, with cisplatin following topotecan as the least toxic sequence, both administration sequences were evaluated. Since, in the 3-weekly schedules combining cisplatin and topotecan, considerable dose reduction of topotecan was necessary, compared with single agent dose, we also studied the possible myeloprotective effect of co-administration of CrEL in scheme C. CrEL was used since in vitro studies indicate that CrEL selectively inhibits cisplatin accumulation in white blood cells, but not in tumour cells (Ma et al, 1996; de Vos et al, 1997). This effect might have been responsible for improvement of therapeutic index observed in a combination study with paclitaxel (van der Burg et al, 1998).

In the current study, in this quite heavily pretreated population, haematological toxicity was the main dose-limiting side effect. However with no patients experiencing febrile neutropenia or

© 2001 Cancer Research Campaign 

requiring platelet transfusions because of bleeding, the observed haematological toxicity was, albeit dose-limiting, relatively easy manageable. Nevertheless dose delays were needed, thereby limiting the projected dose-intensity in scheme A. Since the projected dose-intensity could already not be achieved at the first dose, lower dose was not studied. Also, using the same dose of topotecan studied in scheme A, in the theoretically less toxic sequence of cisplatin following topotecan (scheme B) resulted in 2 out of 2 patients experiencing DLT at the first dose level. Therefore, this schedule was also considered non-feasible. The fact that topotecan was given on the day of cisplatin administration in both schedules, with the only difference consisting of an additional administration of topotecan on the day preceding or following cisplatin, may have accounted for the lack of the initially expected sequence-dependent reduction of haematological toxicity in scheme B. As expected, the drug sequence had no apparent influence on the severity and frequency of non-haematological side effects. These side-effects were mostly mild and mainly consisted of nausea, vomiting, alopecia, headache and neurotoxicity.

With the addition of CrEL in scheme C, no DLTs were observed at the first dose level of topotecan at 0.45 mg $m^{-2}$ and all patients completed the induction without delays, in contrast to the observation in scheme A and B. A dose escalation of 33% for topotecan however again resulted in 2 DLTs in the first 3 patients because of treatment delay for more than 2 weeks due to persistent haematological toxicity. The non-haematological side-effects in scheme C were comparable to those observed for the other 2 schemes. The number of patients in this study was too small to draw unambiguous conclusions on CrELs myeloprotective effect for this combination. However the fact that (1) no haematological DLTs were seen in scheme C compared to 4 out of 10 patients at the same dose level in scheme A, (2) all patients treated on the first dose level of scheme C completed the cycles without delay, and (3) 11 and 0% of cycles in scheme C resulted in grade 3 or 4 neutropenia and thrombocytopenia, as compared to respectively 21 and 9% for scheme A at the same dose level, may all at least suggest a myeloprotective effect of CrEL. The fact that, nevertheless, a dose escalation in scheme C was impossible is likely related to the fact that preclinical data indicate a myeloprotective effect of CrEL on cisplatin toxicity but not on topotecan toxicity, while the administered topotecan might have accounted for most of the haematological toxicity. A randomised study in a patient population treated with a cisplatin-based regimen would be required to further elucidate CrELs potential myeloprotective effect in patients.

Pharmacokinetic analysis revealed no interaction between the compounds in this combination regimen and the apparent clearance of topotecan was independent of dose and schedule. CrEL did not affect the pharmacokinetics of the cytotoxic agents.

The scheduled cisplatin dose intensity of the patients completing all 6 weekly cycles of cisplatin, calculated over a treatment period of 8 weeks was 52.5 mg $m^{-2}$ $week^{-1}$. The achieved median dose intensity of patients completing all 6 weekly induction cycles was 50.5 mg $m^{-2}$ $week^{-1}$ for scheme A; 52.5 mg $m^{-2}$ $week^{-1}$ for scheme B; 60 mg $m^{-2}$ $week^{-1}$ for scheme C first dose level; and 39.5 mg $m^{-2}$ $week^{-1}$ for scheme C second dose level. In comparison to the cisplatin dose intensity 17–25 mg $m^{-2}$ $week^{-1}$ achieved with the standard 3-weekly cisplatin/oral topotecan combination regimens (de Jonge et al, 2000; Gelderblom et al, 2000b), the currently achieved dose intensity of cisplatin in this study is still much higher with all schemes. It is important to note however, that similar dose-intensity of cisplatin can be also achieved in combination with agents such as paclitaxel where the paclitaxel dose-intensity is relatively higher than the currently feasible topotecan dose intensity. The overall 11% of patients withdrawn from the induction regimen due to an allergic reaction to cisplatin is not higher than reported in the literature for highly cisplatin-pretreated populations (Ciesielski-Carlucci et al, 1997). Notably, no allergic reactions were observed in the patients treated with weekly cisplatin preceded by CrEL.

The response rate of 60% in this heavily pretreated population with a relatively short cisplatin-free interval is interesting, but not better compared to weekly cisplatin regimens with agents that can more easily added than topotecan (van der Burg et al, 1996, 1998).

In conclusion, this study suggests, but does not prove, some myeloprotective effect of CrEL on cisplatin-induced toxicity. However a weekly schedule of cisplatin with oral topotecan seems less attractive than a weekly combination of cisplatin with either paclitaxel or etoposide in recurrent ovarian cancer.

### REFERENCES

Badary OA, Abdel-Naim AB, Khalifa AE and Hamada FMA (2000) Differential alteration of cisplatin cytotoxicity and myelotoxicity by the vehicle Cremophor EL. *Naunyn-Schmiedeberg Arch Pharmacol* **361**: 339–334

Chou TC, Motze RJ, Tong Y and Bosl GJ (1994) Computerised quantitation of synergism and antagonism of Taxol, Topotecan and Cisplatin against human teratocarcinoma cell growth: a rational approach to clinical protocol design. *J Natl Cancer Inst* **86**: 1517–1524.

Ciesielski-Carlucci C, Leong P and Jacobs C (1997) Case report of anaphylaxis from cisplatin/paclitaxel and a review of their hypersensitivity reaction profiles. *Am J Clin Oncol (CCT)* **20**: 373–375

Creemers GJ, Bolis G, Gore M, Scarfone G, Lacave AJ, Guastalla JP, Despax R, Favalli G, Kleinberg R, van Belle S, Hudson I, Verweij J and ten Bokkel Huinink WW (1994) Topotecan, an active drug in the second line treatment of epithelian ovarian cancer: Results of a large European Phase II study. *J Clin Oncol* **12**: 1748–1753

de Jonge MJA, Loos WJ, Gelderblom HJ, Planting AST, van der Burg MEL, Sparreboom A, Brouwer E, van Beurden V, Mantel M, Doyle E, Hearn S, Ross G and Verweij J (2000) Phase I and pharmacological study of oral topotecan and intravenous cisplatin: sequence-dependent haematologic side effects. *J Clin Oncol* **18**: 2104–2115

de Vos Al, Nooter K, Verweij J, Loos WJ, Brouwer E, de Bruijn E, Ruijgrok EJ, van der Burg MEL, Stoter G and Sparreboom A (1997) Differential modulation of cisplatin accumulation in leukocytes and tumour cell lines by the paclitaxel vehicle Cremophor EL. *Ann Oncol* **8**: 1145–1150

Gelderblom H, Loos WJ, Verweij J, Brouwer E, van der Burg MEL and Sparreboom A (2000a) Modulation of cisplatin pharmacodynamics by Cremophor EL: Experimental and clinical studies. *Submitted*

Gelderblom H, Loos WJ, de Jonge MJA, Sparreboom A, Planting AST, van der Burg MEL, Brouwer E, Verheij C, Ouwens L, Hearn S and Verweij J (2000b) Phase I and pharmacological study of increased dose oral topotecan in combination with intravenous cisplatin. *Ann Oncol* **11**: 1205–1207

Gerrits CJH, Burris H, Schellens JHM, Planting AST, van der Burg MEL, Rodriguez Gl, van Beurden V, Loos WJ, Hudson I, Fields S, Verweij J and Von Hoff D D (1998) Five days of oral topotecan (Hycamtin), a phase I and pharmacological study in adult patients with solid tumours. *Eur J of Cancer* **34**: 1030–1035

Gore M, Rustin G, Calvert H, Bezwoda W, Carmichael J, Oza A, Kaye S, ten Bokkel Huinink W, Malfetano J, Falkson G, Clarke-Pearson D, Ross GA, Dane GC and Fields SZ (1998) A multicentre randomized phase III study of topotecan administered intravenously or orally for advanced epithelial ovarian carcinoma. *Proc ASCO* **17** (abstr.): 1346

Hochster H, Liebes L, Speyer J, Sorich J, Taubes B, Oratz R, Wernz J, Chachoua A, Blum RH and Zeleniuch-Jacquotte A (1994) Phase 1 trial of low continuous topotecan infusion in patients with cancer: An active and well tolerated regimen. *J Clin Oncol* **12**: 553–559

Liu G, Franssen E, Fitch MI and Warner E (1997) Patient preferences for oral versus intravenous palliative chemotherapy. *J Clin Oncol* **15**: 110–115

Ma J, Verweij J, Planting AST, Kolker HJ, Loos WJ, de Boer-Dennert M, van der Burg MEL, Stoter G and Schellens JHM (1996) Docetaxel and paclitaxel inhibit DNA adduct formation and intracellular accumulation of cisplatin in human leukocytes. *Cancer Chemother Pharmacol* **36**: 382–382

 *© 2001 Cancer Research Campaign*

McGuire WP and Ozols RF (1998) Chemotherapy of advanced ovarian cancer. *Semin Oncol* **25**: 340–348

Miller AA, Hargis BJ, Lilenbaum RC, Fields SZ, Rosner GL and Schilsky RL (1994) Phase 1 study of topotecan and cisplatin in patients with advanced solid tumours: a cancer and leukemia group B study. *J Clin Oncol* **12**: 2743–2750

Parker SL, Tong T, Bolden S and Wingo PA (1997) Cancer statistics, 1997. *CA Cancer J Clin* **47**: 5–27

Romanelli S, Perego P, Pratesi G, Carenini N, Tortoreto M and Zunino F (1998) *In vitro* and *in vivo* interaction between cisplatin and topotecan in ovarian carcinoma systems. *Cancer Chemother Pharmacol* **41**: 385–390

Rowinsky EK, Kaufmann SH, Baker SD, Grochow LB, Chen T-L, Peereboom D, Bowling MK, Sartorius SE, Ettinger DS, Forastiere AA and Donehower RC (1996) Sequences of topotecan and cisplatin: Phase 1, pharmacologic, and in vitro studies to examine sequence dependence. *J Clin Oncol* **14**: 3074–3084

Swisher EM, Mutch DG, Rader JS, Elbendary A and Herzog TJ (1997) Topotecan in Platinum-and Paclitaxel-resistant ovarian cancer. *Gyn Oncol* **66**: 480–486

ten Bokkel Huinink W, Gore M, Carmichael J, Gordon A, Malfetano J, Hudson I, Broom C, Scarabelli C, Davidson N, Spanczynski M, Bolis G, Malmstrom H, Coleman R, Fields SC and Heron JF (1997) Topotecan versus paclitaxel for the treatment of recurrent epithelial ovarian cancer. *J Clin Oncol* **15**: 2183–2193

van der Burg MEL, Logmans A, de Wit R, van Lent M, Kruit WJH, Stoter G and Verweij J (1996) Weekly high dose cisplatin and oral vepesid: a highly active regimen for ovarian cancer failing on or relapsing after conventional platinum containing combination chemotherapy. *Proc ASCO* **15** (abstract): 722

van der Burg MEL, de Wit R, Stoter G and Verweij J (1998) Phase 1 study of weekly cisplatin and weekly or 4-weekly taxol: a highly active regimen in advanced epithelian cancer. *Proc ASCO* **17** (abstr): 1370

*© 2001 Cancer Research Campaign*
